# DB-AT: a 2015 update to the Full-parasites database brings a multitude of new transcriptomic data for apicomplexan parasites

**DOI:** 10.1093/nar/gku1240

**Published:** 2014-11-20

**Authors:** Marcin Jąkalski, Hiroyuki Wakaguri, Tabea G. Kischka, Yoshifumi Nishikawa, Shin-ichiro Kawazu, Makoto Matsubayashi, Fumiya Kawahara, Naotoshi Tsuji, Shinuo Cao, Fujiko Sunaga, Xuenan Xuan, Kazuhiro Okubo, Ikuo Igarashi, Josef Tuda, Arthur E. Mongan, Yuki Eshita, Ryuichiro Maeda, Wojciech Makałowski, Yutaka Suzuki, Junya Yamagishi

**Affiliations:** 1 University of Münster, Faculty of Medicine, Institute of Bioinformatics, Niels-Stensen Strasse 14, 48149 Münster, Germany; 2 Department of Medical Genome Science, Room 032, Sougou Research Complex, 5-1-5 Kashiwanoha, Kashiwa, Chiba 277-8562, Japan; 3 National Research Center for Protozoan Diseases, Obihiro University of Agriculture and Veterinary Medicine, Obihiro, Hokkaido 080-8555, Japan; 4 National Institute of Animal Health, National Agricultural and Food Research Organization, Kannondai, Tsukuba, Ibaraki 305-0856, Japan; 5 Nippon Institute for Biological Science, 9-2221-1 Shin-machi, Ome, Tokyo 198-0024, Japan; 6 Department of Parasitology, Kitasato University School of Medicine, 1-15-1 Kitasato, Minami-ku, Sagamihara, Kanagawa 252-0374, Japan; 7 School of Veterinary Medicine, Azabu University, 1-17-71 Fucinobe, Chuo-ku, Sagamihara, Kanagawa 252-5201, Japan; 8 Department of Medicine, Sam Ratulangi University, Kampus Unsrat, Bahu Manado, 95115, Indonesia; 9 Oita University, School of Medicine, 1-1 Idaigaoka, Hazamacho, Yufu, Oita 879-5593, Japan; 10 Obihiro University of Agriculture and Veterinary Medicine, Inada-cho West 2-13, Obihiro, Hokkaido 080-8555, Japan; 11 Division of Collaboration and Education, Research Center for Zoonosis Control, Hokkaido University, North 20, West 10 Kita-ku, Sapporo, Hokkaido 001-0020, Japan; 12 Global Station for Zoonosis Control, GI-CoRE, Hokkaido University, North 20, West 10 Kita-ku, Sapporo, Hokkaido 001-0020, Japan

## Abstract

The previous release of our Full-parasites database (http://fullmal.hgc.jp/) brought enhanced functionality, an expanded full-length cDNA content, and new RNA-Seq datasets from several important apicomplexan parasites. The 2015 update witnesses the major shift in the databases content with focus on diverse transcriptomes of the apicomplexan parasites. The content of the database was substantially enriched with transcriptome information for new apicomplexan parasites. The latest version covers a total of 17 species, with addition of our newly generated RNA-Seq data of a total of 909 150 388 tags. Moreover, we have generated and included two novel and unique datasets, which represent diverse nature of transcriptomes in individual parasites *in vivo* and *in vitro*. One is the data collected from 116 Indonesian patients infected with *Plasmodium falciparum*. The other is a series of transcriptome data collected from a total of 38 single cells of *P. falciparum* cultured *in vitro*. We believe that with the recent advances our database becomes an even better resource and a unique platform in the analysis of apicomplexan parasites and their interaction with their hosts. To adequately reflect the recent modifications and the current content we have changed the database name to DB-AT—DataBase of Apicomplexa Transcriptomes.

## INTRODUCTION

The Apicomplexa group of protozoans consists of many parasite species that are responsible for bringing numerous serious health risks worldwide (1–4). A detrimental effect of apicomplexan parasites is not limited to human health issues only but has a huge economical effect as well. For instance, there is an estimated annual loss of a few billion USD in global poultry farming caused by coccidiosis (5,6). Chemicals and vaccinations, however scarce, are currently the major control agents for the Apicomplexa-caused diseases. Nonetheless, frequent emergences of parasites resistant to treatment become a serious problem. A good resource is needed that could serve as a universal platform for understanding the underlying mechanism and principles of parasitism of Apicomplexa species. To this end, a great potential has already been proved to lie in genome sequences and gene expression information. The latter one especially serves as a powerful analysis tool for connecting genotype to phenotype information. The advent of the Next Generation Sequencing (NGS) technologies has made a substantial contribution in this field and now a so-called RNA-Seq method (7) had been proved to work even on a very small, such as a single cell scale (8). Indeed, there are numerous examples in which RNA-Seq technology was used for distinguishing gene splice variants, validation of already annotated transcripts and as well as identification of novel ones, both in model and non-model organisms.

With this 2015 update, we intended to enrich the transcriptomic data of apicomplexan parasites mainly in two aspects. Firstly, the current version includes new transcriptome data for eight apicomplexan parasites, namely *Plasmodium berghei* (*Pb*), *Theileria equi* (*Te*), *Neospora caninum* (*Nc*), *Eimeria maxima* (*Em*), *Babesia caballi* (*Bc*), *Babesia divergens* (*Bd*), *Babesia bigemina* (*Bb*) and *Babesia gibsoni* (*Bg*). This expanded coverage of the Apicomplexa species should be useful to complement and cross-check the gene repertoire that is already deposited in other publicly available databases such as PlasmoDB (http://plasmodb.org/, (9)), ToxoDB (http://toxodb.org/, (10)), PiroplasmaDB (http://piroplasmadb.org/, (11)), CryptoDB (http://cryptodb.org/, (12)), that are integrated in the EupathDB (http://www.eupathdb.org/, (11)). Secondly, we have included two novel datasets, which were generated based on our recent studies, to reveal the diversity of transcriptomes of *Plasmodium falciparum* (*Pf*), both *in vivo* and *in vitro*. One is a dataset collected from 116 Indonesian clinical samples, in which RNA-Seq data, both of host humans and infecting parasite cells, are simultaneously represented (13). The other is a dataset from single-cell RNA-Seq analysis of an *in vitro* cultured 3D7 strain of *Pf*. It also represents changes of transcriptomes in parasites in response to chloroquine treatment in different time courses (0, 6, 12, 24 and 48 h).

To facilitate an easier access and representation of the newly generated datasets, we additionally applied several improvements to our database. Particularly, we introduced a newly implemented genome viewer which enables flexible control of displayed tracks, each of which representing RNA-Seq data of individual parasites. All of the above-described updates were integrated with the former datasets that were introduced alongside the previous releases of our apicomplexan parasite repository (14–16). Taking into account that we brought together many different types of the deposited data in our database, we decided to change its name to a more generic one, namely DB-AT—DataBase of Apicomplexa Transcriptomes. We believe that our work, as a part of extensive international effort to broaden our knowledge in the field of parasitism and related diseases, will provide a unique platform for further analyses, e.g. selection of candidate causative-genes, detailed understanding of the mechanism behind diseases and aid in designing a successful cure. DB-AT is accessible at http://fullmal.hgc.jp/.

## DATABASE DESCRIPTIONS

### Statistics of the new transcriptome data for additional species

In this update, we extended the content of the database to cover transcriptome sequences of the following additional eight representative species of the Apicomplexa phylum: *Pb*, *Nc*, *Te*, *Em*, *Bg*, *Bc*, *Bb* and *Bd*. By RNA-Seq analysis using Illumina HiSeq 2500, we generated a total of 909 150 388 paired-end 100 bp RNA-Seq tags from different developmental/infectious stages of the aforementioned parasites (Table 1). For the species for which the reference genome sequences have been sequenced, we retrieved the sequences mainly from EupathDB (11) and its integrated databases. See our web page (http://fullmal.hgc.jp/docs/statistics_2015.html) for the source of the primary data. To complement still incomplete genome sequencing or gene annotations for several species, we also assembled transcripts using the RNA-Seq tags. It is clearly visible that, in comparison to the reference genome annotation, a significant proportion of the previously annotated coding loci are represented in our database and at the same time, many novel ones were discovered. Sequencing and assembly of RNA-Seq tags resulted in a total of 5291 transcripts (3986 loci), 6045 transcripts (5183 loci), 12 904 transcripts (7986 loci) and 15 688 transcripts (10 177 loci) from *Pb*, *Te*, *Nc* and *Em*, respectively (see Statistics page of the database). In the DB-AT, the generated transcript models can be compared to the respective reference genome annotations from other public databases like PlasmoDB, ToxoDB and PiroplasmaDB through our genome viewer, as well as used for investigating unique transcriptome repertoires specific to given cell stages of infecting parasites. For the species without a reference genome available, *de novo* assembly was performed. We obtained 18 687, 13 490, 12 474 and 15 892 putative transcript sequences from *Bc*, *Bd*, *Bg* and *Bb*, respectively (Table 1). Now, DB-AT covers transcriptome data for a total of 17 Apicomplexa species.

**Table 1.** Statistics for new RNA-Seq tags of apicomplexa parasites

Species	Strain	Stage	eference	Total sequenced tags	Mapped tags (%)	No. of represented transcripts^c^
*Plasmodium berghei*	ANKA	Merozoite	PlasmoDB-11.0	142 359 734	91.2^a^	3108
*Theileria equi*	USDA	Merozoite	PiroplasmaDB-5.0	117 015 108	23.0^a^	4018
*Neospora caninum*	Nc-1	Tachyzoite	ToxoDB-11.0	117 043 870	83.1^a^	8160
*Eimeria maxima*	NIAH	Mature oocyst	ToxoDB-11.0	68 834 980	85.2^a^	8619
		Sporozoite		100 289 968	84.0^a^	
*Babesia caballi*	USDA	Merozoite	NA	80 656 968	95.63^b^	18 687
*Babesia divergens*	Undetermined	Merozoite	NA	89 996 032	93.93^b^	13 490
*Babesia gibsoni*	Oita	Merozoite	NA	73 740 894	97.91^b^	12 474
*Babesia bigemina*	Argentina	Merozoite	NA	119 212 834	94.87^b^	15 892

^a^Mapped to the reference genome.

^b^Mapped back to assembled transcripts.

^c^FPKM > 1.

### New RNA-Seq datasets of diverse transcriptome features of *Plasmodium falciparum*

**Figure 1.**
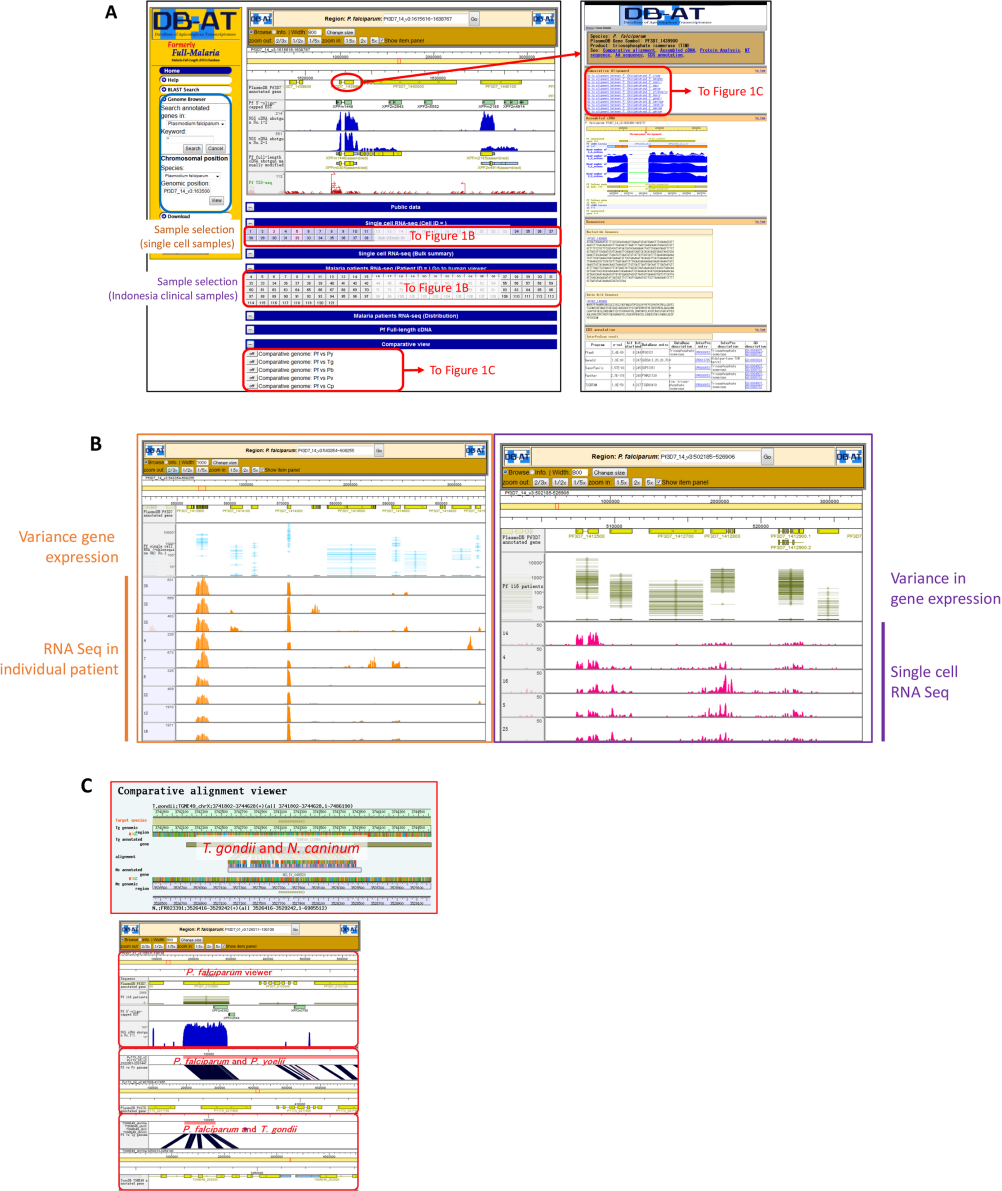
(**A**) Updated genome viewer of the DB-AT. To search the database users can select species of interest and gene name/identifier. Alternatively, keyword searches are also possible, as well as direct jump to a known genomic position (left upper blue box). Available tracks can be selected from below the genome browser. The screen shot shows a case of the PF3D7_1439900 gene. Positions indicated in orange, purple and red boxes are links to the results of single cell analysis—Indonesian clinical samples and Comparative view, respectively. Toggling the ‘Browse’ to ‘Info’ button and subsequent click on the desired gene gives access to the additional information about each gene. Drawn arrows point various viewers, which can be followed from the indicated items. (**B**) Viewers of Single cell/Indonesian samples, to examine divergent expression patterns. (**C**) Gene Annotation Viewer, in which functional annotations of the indicated genes is represented (middle), and Comparative Genomics Viewer. Further information on search conditions, available tracks, and other database features can be found in the ‘Help’ section (http://fullmal.hgc.jp/fm_help_2015.html).

Recently, we have conducted a single cell parasite RNA-Seq analysis using an *in vitro* culture of *Pf* strain 3D7. Analysis of the generated RNA-Seq tags revealed surprisingly diverse patterns of gene expression between individual parasites, which were cultured in a uniform culture conditions (see Figure 1B for an example). To further monitor how those observed transcriptome diversities of the 3D7 strain were altered by the administration of an anti-malaria drug, chloroquine, we similarly generated a series of single parasite RNA-Seq libraries in a time course manner after the drug treatment (0, 6, 12, 24 and 48 h). A total of 89 533 883 RNA-Seq tags were collected from 246 single parasites in all the culture conditions taken together and were integrated into current database update (for details see Table 2A). Further details on the biological characterization of the observed divergence will be published elsewhere.

**Table 2.** Population diversities in *Plasmodium falciparum*

**A.** Statistics for single-cell datasets of *P. falciparum* in five time courses						
Hours after chloroquine treatment	Number of cell samples	Number of mapped tags				
0 h (replicate 1)	38	27 623 157				
0 h (replicate 2)	38	27 700 140				
6 h	45	26 080 027				
12 h	37	6 130 583				
24 h	41	1 541 863				
48 h	47	458 113				

**B.** Clinical samples of Indonesian malaria patients						
Species	Genome version	Datasets	Total mapped tags	Average frequency of parasite tags (%)	Mapped tags	No. of represented genes (RPKM > 1)
*P. falciparum*	PlasmoDB-11.0	116	3 066 215 421	9.1	244 993 536	3569
Human	UCSC hg38				2 846 381 177	8844

Another unique aspect of this update is the clinical transcriptome data from clinical malaria samples. This datasets covers a total of 116 samples of Indonesian patients infected with malaria parasite. The blood samples were subjected to the RNA-Seq analysis as a mix, so that transcriptome information of host humans and infecting parasites should be obtained simultaneously. As expected, the analysis yielded approximately 90% of the RNA-tags from humans and the rest was from parasites. Essentially, no tags mapped to both human and parasite genomes. Several parasite genes whose expression levels were positively or negatively correlated to those of the host human genes were identified. Further details of this study has been already published elsewhere (13) and a summary of the dataset is present in Table 2B. To analyze a mutual relationship between transcriptome features of humans and parasites identified from the same samples, users can now view the transcriptome information both for humans and parasites. We believe such ‘interactive’ transcriptome analysis will reveal how diverse expression patterns, both in humans and parasites, will eventually lead to different clinical malaria symptoms.

### DB-AT features

Taking advantage of the newly added RNA-Seq data and assembled sequences, along with the unique Full-length cDNAs, Transcription Start Site (TSS) tags, and EST data known from previous rounds of updates, various kinds of transcript-based analyses are possible. Since many users were already familiar with the database and the functions it offers and to avoid confusion, we decided to keep the overall design and functionality unchanged. As is the case of the previous version, users can search the respective repositories by using keywords, e.g. gene IDs or annotated function, or by specifying desired genomic positions (Figure 1A). The major improvements have been done to the genome viewer. To allow more interactive access to the stored datasets, a new HTML5 canvas has been implemented. It supports easy scrolling along the chromosome/scaffold length and easy in/out zooming. Settings for the displayed tracks can now be accessed from below the main viewer window. All the main track categories, like public data, full-length cDNA, TSS-seq, etc., are displayed as collapsible menus for more effective management of the workspace. A ‘Comparative view’ to examine evolutionary conservation of transcript sequences and proximal regions is also available as a track for some species (Figure 1C). The alignment of the transcript can be also followed from the ‘Annotation’ viewer of the associated transcript of a closely-related species (Figure 1A, right panel). In addition to the previous tracks available, new tracks appear for the newly generated RNA-Seq data and the transcriptome assemblies. Users can check the overall coverage and the quality of the assembled transcripts from the RNA-Seq tags pileup information, view the predicted splice junctions, and see how the generated RNA-Seq tags are distributed along the genome. The biggest change is visible in case of dynamic transcriptome data from *Pf* samples and related experiments. The expanded set of available tracks that can be added contains information from all individual samples. The available summary tracks show divergence in the expression levels of each transcript in the given dataset. In case of the clinical samples on malaria patients, an additional feature was implemented. Where the information on the human gene expression is available as a counterpart for each *Pf* sample, a direct link is added to jump to the new Human viewer. Moreover, most of the items in each available track can be clicked to modify their display modes and to access additional information including, e.g. nucleotide sequences or a link to the reference database (Figure 1A).

Additionally, this version of our database complies with the most recent versions of the reference databases, namely PlasmoDB (http://plasmodb.org/plasmo/, Release 11.0), ToxoDB (http://toxodb.org/toxo/, Release 11.0), PiroplasmaDB (http://piroplasmadb.org/piro/, Release 5.0), and CryptoDB (http://cryptodb.org/cryptodb/, Release 6.0). Moreover, the BLAST searches available from the database website now feature tBLASTn program to facilitate usage of protein queries.

### Search example

To present the functionality offered by our updated database, Figure 1 exemplifies the case of a *Pf* gene annotated as triosephosphate isomerase (PF3D7_1439900), which plays an important role in metabolic pathway of *Pf*. Particularly, diverse expression levels were observed for this gene both among 116 Indonesian clinical samples and *in vitro* cultured individual cells. Expression (bottom left panels; Figure 1B) of this gene has been recently proved to be significantly associated with the increased body temperature in the malaria-infected patients (13). Go to the home page and select *P. falciparum* in the ‘Search annotated genes in’ box. As a keyword for search specify ‘PF3D7_1439900′. Follow the ‘Genomic position’ link from the search results page to view the gene in its genomic neighborhood. By exploring the tracks present at the bottom of the browser (all track sections collapsed by default) users can, for example, observe fading expression values of the gene from the single-cell RNA-Seq experiment after the application of chloroquine treatment (five different time courses) or explore expression profiles of selected individuals out of 116 Indonesian malaria-infected patients. Additionally, by changing the radio button from ‘Browse’ to ‘Info’ in the top-left corner of the browser, and by subsequent mouse click on the gene, users can access further information about it, including nucleotide and amino acid sequence, a link to the reference database, etc.

### Data access and further information

Detailed information on the database features and usage are described in the ‘Help’ section of our database. Statistics for the database repositories (including previous releases) are available in the ‘Statistics of this Database’ section. The newly generated datasets, including raw RNA-Seq reads, are freely available without any restriction from the ‘Download’ section of our database (http://fullmal.hgc.jp/cgi-bin/download/). Data from previous releases can also be accessed from the same resource.

## CONCLUSIONS AND FUTURE PERSPECTIVES

Here, we have described all the major updates to our apicomplexan parasites database that has been renamed from Full-parasites to DB-AT. The previous content has been expanded with extensive RNA-Seq data from many different species and experiment types. A dynamic nature of transcriptomes, with each cell type, stage and conditions, and above all, different individual parasites, expressing a unique ensemble of transcripts, brings a tremendous diversity. We have put an extensive effort to collect as much of such dynamic data as possible and deposited it in our database, making it a unique resource among other parasite-oriented databases. We believe that our unique dataset should deserve to be further evaluated and utilized by the users who investigate diverse expression patterns of parasites, thus, should serve as a complementary database to the pivotal databases, such as EupathDB, PlasmoDB and alike. In the future, we plan to continue to expand the content of our database by adding new transcriptome data from other species, as well as the datasets representing how diverse transcriptome features may lead to a diverse biology of apicomplexan parasites, regarding their interactions with their host cells, complex life cycles and drug sensitivities.

## SUPPLEMENTARY DATA

Supplementary Data are available at NAR Online.
